# A Survey on the Evolution of Opportunistic Routing with Asynchronous Duty-Cycled MAC in Wireless Sensor Networks

**DOI:** 10.3390/s20154112

**Published:** 2020-07-23

**Authors:** Ayesha Akter Lata, Moonsoo Kang

**Affiliations:** Department of Computer Engineering, Chosun University, 375 Seosuk-Dong, Dong-Gu, Gwangju 501-759, Korea; lata_sust@yahoo.com

**Keywords:** wireless sensor networks, asynchronous duty-cycled mac, opportunistic routing protocols, duty cycle, load-balancing, energy consumption, asynchronous WSN

## Abstract

Wireless sensor networks (WSNs) have been used for environmental monitoring and reporting for many decades. Energy consumption is a significant research topic because wireless sensor nodes are battery-operated to be highly energy-constrained. Several strategies have been introduced in routing and MAC (Medium Access Control) layer protocols to facilitate energy saving. At the routing layer, an energy-efficient routing protocol, known as opportunistic routing (OR), has been designed to improve efficiency. OR achieves energy efficiency via load-balancing, which forwards packets along multiple routes over WSNs. At the MAC layer, an energy-efficient MAC protocol known as the asynchronous duty-cycled MAC (ADCM) protocol achieves energy saving by turning on and off a sensor node’s transmitter and receiver to eliminate unnecessary energy wastage. These protocols each have their own advantages and disadvantages. OR achieves energy efficiency at the routing layer but it raises an issue at the MAC layer. ADCM achieves energy efficiency at the MAC layer, but it hinders the packet forwarding efficiency of the OR. To attain better energy efficiency, a combination of these two ideas led to the development of OR with asynchronous duty-cycled MAC (OR-ADCM). However, even with better energy efficiency, limitations still exist in combining load-balancing and duty-cycling due to conflicts in the inherent properties of OR and ADCM. In this paper, we present a survey of the evolution of OR-ADCM over WSNs to help the reader better understand and appreciate the details of this tradeoff, which we hope will lead to the development of better protocol designs.

## 1. Introduction

Wireless communication has made huge contributions to modern technology and information communication. Since the development of this technology, numerous protocols have been proposed to improve communication performances, and several other protocols continue to be introduced. To make new improvements, it is necessary to gain an in-depth understanding of previous and current issues and trends. In this paper, we have highlighted the significant improvements made in the area of wireless sensor networks (WSNs) regarding the routing mechanisms underlying energy-constrained sensor nodes. Our main focus is the ability of WSNs to balance energy consumption loads across battery-operated sensor nodes in order to extend network lifetime.

The duty cycle [1] mechanism, which was introduced to reduce the energy consumption of sensor nodes, is arguably one of the greatest milestones in the development of WSNs. When one node performs packet transmission, other nodes in its neighborhood hold themselves from transmitting packets that are unnecessary for them, and silently listen to the ongoing transmission. This idle listening results in a significant waste of energy. Although the duty cycle technique can reduce unnecessary energy consumption, it also introduces additional issues [2]. A node needs to know when the neighbor node wakes up in order to start its transmission, and for that the network falls into a time domain. Nodes using duty-cycling must use a synchronization mechanism that coordinates neighboring sensor nodes for smooth transmission. Nonetheless, multi-hop network time synchronization leads to a high control packet overhead, and border nodes have to match their duty cycle according to their neighbor nodes, which extends the duration of active time. Moreover, the energy consumption during synchronous duty-cycling increases sharply with one or multiple communications and parallel traffic flows, and as a result, synchronization does not scale well in large and condensed networks. On the contrary, the asynchronous duty cycle mechanism does not require any previous synchronization. During a less congested traffic load, a large number of available asynchronous MAC protocols minimizes energy consumption. Hence, using an asynchronous duty cycle over a synchronous mechanism in a WSN is more energy-efficient.

Because our concern is in minimizing the energy consumption of sensor nodes, we consider traditional unicast routing to be too expensive for duty-cycled WSNs. In conventional unicast routing, nodes must wait for the destined node until it wakes up, which costs additional energy and time. On the other hand, opportunistic routing chooses its receiver opportunistically and provides dynamic routing progress, which is both energy and time efficient. In this study, we focused on advancements in the area of opportunistic routing with asynchronous duty-cycled WSNs. First, we prefer opportunistic routing over unicasting in asynchronous duty-cycled WSNs in order to decrease the energy consumption load on the sender node while it waits for the predefined forwarder node to wake up and receive the packet. Using other available routes for sending packets instead of waiting longer times for one specific node is more energy-efficient. Moreover, using different routes for different transmissions can also balance the load distribution of the energy-constrained WSNs. However, two major issues arise with this method. First, multiple forwarders wake up at the same time, and as a result, there is a high probability of collisions occurring among them while attempting to forward the same packet to the next hop. Second, the duplicate packet problem will result in unnecessary energy consumption. Many studies have been conducted to resolve these issues. The main objective of these protocols is to minimize the size of the forwarder set so that only the best forwarder node can forward the packet effectively and help the transmission run smoothly.

In addition to the above-mentioned issues, there are several other issues that have been discussed in the literature [3] and that we address in this review, such as data redundancy, dynamic route selection, forwarder cooperation, etc. (For more details, refer to the section of “Issues of Opportunistic Routing in Asynchronous WSN”). We highlight some important concerns brought up by our survey, and review recent developments in solving these problems. We further describe the basic concepts underlying the opportunistic routing protocol, point out important issues related to opportunistic routing when applied to asynchronous WSNs, discuss recent advancements, and provide a comparison between these advancements.

## 2. Related Studies

In this section, we present an at-a-glance review of studies that are related to the evolution of opportunistic routing protocols with asynchronous duty-cycled MAC in WSNs. WSNs, in which nodes share a single channel or multiple channels and contend for their transmission, have both advantages and disadvantages. Opportunistic routing, on the other hand, uses the shared channel characteristic as an advantage with regards to forwarding data. Using the broadcasting mechanism, it searches for available and suitable nodes dynamically in order to forward the data to the target sink node. In this way, this protocol can distribute the energy load from one node to other usable nodes. In a dense network, where it is costly to maintain a topology and to run synchronization processes repeatedly, opportunistic routing with asynchronous duty cycles plays a significant role.

**Figure 1:**
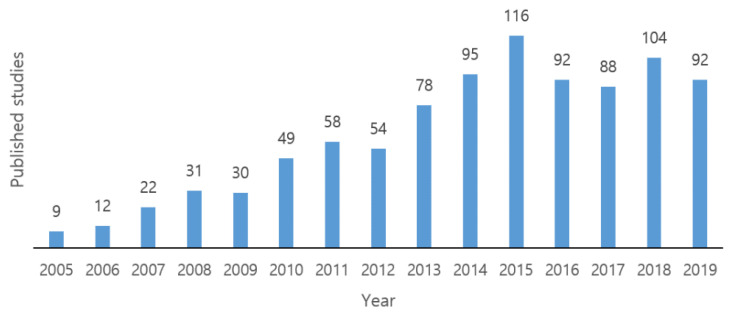
Statistics on the publication related to OR with ADCM in WSNs.

The concept of opportunistic routing, also known as ExOR, was first proposed by Biswas and Morris in 2005 [4]. This protocol proved to be robust, increasing throughput by almost 35% compared to conventional protocols. Nevertheless, the authors only presented simulations describing the throughput, but did not discuss the issue of energy consumption. The advantages of OR grabbed the attention of researchers as it would now be possible to improve loopholes and make routing more energy-efficient, thus boosting the performance of WSNs. Subsequently, using the principles of ExOR, many other opportunistic routing protocols have been proposed [5,6,7,8,9,10]. However, these protocols assume that the sensor nodes are always active and do not consider the duty-cycle mechanism for energy efficiency. Hence, several other protocols [11,12,13,14,15,16,17] have been proposed, where opportunistic routing is combined with duty-cycled MAC in order to prevent unnecessary energy consumption. These protocols were significant developments in the opportunistic routing paradigm for asynchronous WSNs, and successfully balanced network load with extended lifetime efficiently. Figure 1 presents the sustained developments in terms of the number of publications every year related to OR with ADCM over WSNs. From the site, scholar.google.com, we have searched the related papers on the topic of OR with ADCM with the key words and expressions “opportunistic routing” + asynchronous + duty + cycle + mac + wireless + sensor + network. We counted only the number of published studies since 2005, because the first duty cycle MAC, S-MAC, was published in 2002, and the first opportunistic routing protocol, ExOR, was published in 2005.

Table 1 shows an at-a-glance summary of survey articles on OR and MAC protocols related to our study. To gain a better idea of the taxonomy of opportunistic routing protocols, we refer readers to a previous article [18]. In the mentioned article [18], the authors have explained in detail the types of opportunistic routing protocols and their characteristics. The authors have also provided a comparative analysis that can provide a clear view of the main features of the protocols and their contributions. However, the authors in [18] have only focused on overall opportunistic routing protocols based on their structure and working process, without detailing energy consumption factors. Because of their useful characteristics, studies have been conducted to evaluate the performance of opportunistic routing protocols in other communication networks as well [19].

Moreover, to appreciate the importance of combining routing with duty-cycled MAC protocols in order to reduce the energy consumption of the sensor node, readers can refer to the survey by Nithya and Mahendran [20]. In [20], the authors have described different types of scheduling methods for duty-cycling in the MAC layer and routing schemes, including opportunistic routing. They analyzed protocols and presented their respective pros and cons to show their effectiveness with regard to energy efficiency. However, they did not provide a brief review nor a comparison of protocols in the literature.

Patel and Kamboj, 2015 [21], focused on the issue of reducing retransmission in order to prevent unnecessary energy consumption. Similar to Chakchouk, 2015 [18], the paper by Patel and Kamboj [21] also described the main design blocks of opportunistic routing protocols. However, they divided the protocol into two main parts: (i) coordination method and (ii) candidate selection method. Since their main focus was on taking control over retransmission, they reviewed protocols on the basis of their candidate selection processes and coordination schemes. As we know, by structure, opportunistic routing protocols run their processes depending on the MAC protocol used by the node. In contrast, the authors did not specifically mention the underlying MAC mechanism of the reviewed OR protocols.

In Sharma et al., 2014 [3], the authors reviewed a number of opportunistic routing protocols, wherein they focused on all WSN issues and discussed the contribution of the opportunistic routing protocol to these issues. However, they excluded a crucial point by not discussing the MAC mechanism underlying the protocols. Moreover, the protocols reviewed in the paper were assumed to be active all the time, which means they did not take into account the duty-cycle.

**Figure 2:**
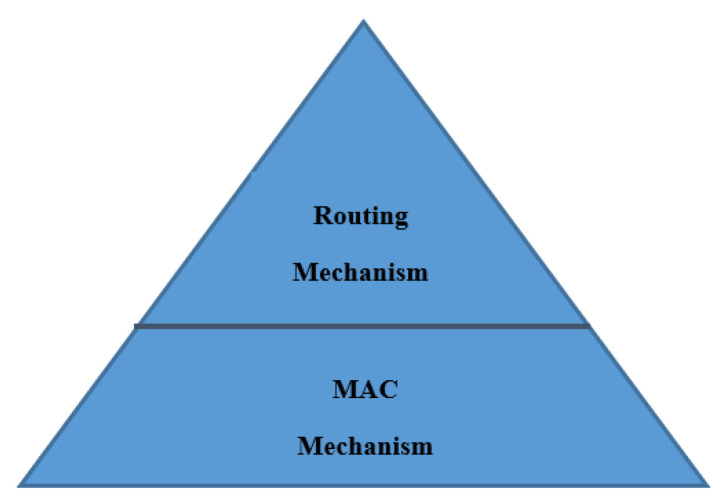
Decision-making hierarchy of opportunistic routing protocol.

On the other hand, all opportunistic routing protocols have been established based on a specific MAC protocol. The routing decision they made for their process depends on the underlying MAC protocol because, since the beginning, the main concept of opportunistic routing has been a combination of MAC and a routing mechanism, as represented in Figure 2. Several survey articles [22,23,24,25,26,27] have studied the existing MAC protocols in the literature, focusing on their contributions to preserving the energy and lifetime of the network.

Yahya et al. [22] surveyed MAC protocols in the literature, focusing on the energy efficiency, latency and throughput factors of WSNs. This paper classified the existing MAC protocols based on the underlying mechanisms they worked with. Depending on classification characteristics, they covered a large number of protocols and have explained them specifically with regard to different aspects.

Bachir et al. [23] mainly classified protocols focusing on the WSN issues that they were working on. This article covers several protocols and explains their functionalities in order to solve these issues. This survey also points out some of the hardware factors of the protocols. It may therefore help readers obtain a brief view of almost all the types of characteristics of the protocols.

Huang et al. [24] not only classified protocols, but also explained the course of motivation that drove the evaluation of the protocols. This paper points out the necessity and characteristics of the issues and solutions that these protocols were trying to address. The main focus of the asynchronous MAC protocols discussed here was time management and the sender and receiver waiting time.

Kakria et al. [25] conducted a study on the latest asynchronous MAC protocols at the time, and explained how sensor nodes communicate with each other using their mismatched duty-cycles. They mainly focused on the energy consumption factors of the protocols.

Alfayez et al. [26] discussed both synchronous and asynchronous MAC protocols and their respective issues in the MAC layer for WSNs. Here, the authors examined their performances considering a linear/chain-type WSN.

Anubhama et al. [27] reviewed MAC protocols based on throughput, efficiency, stability, fairness, low access delay, low transmission delay and low overhead. The article did not specifically focus on duty-cycling or synchronous and asynchronous characteristics; rather, it evaluates the protocols by taking into account the specifications mentioned above.

Thus, each previous study either reviewed only opportunistic routing protocols or only MAC protocols. They did not provide any survey discussing both protocols, and did not describe their compatibility and adjustments to each other while focusing on preserving energy. In this study, we aimed to discuss energy efficiency, and fixed our focus on the load-balancing of asynchronous duty cycles combined with opportunistic routing. Hence, protocols related to this specific issue are described here. Among them, ORW [11], ORD [12], ORR [13], MOR [14], MORR [15], LORA [16] and LEOR [17] are arguably the most significant opportunistic routing protocols that have efficiently improved the aforementioned issues, while combining with unscheduled duty-cycled MAC protocols and balancing energy consumption effectively. Additionally, we have provided a review of the evolution of asynchronous duty cycle MAC protocols in WSNs from their early years to the present time. Instead of surveying a large number of protocols, we have described a few significant modified asynchronous MAC protocols [28,29,30,31,32,33,34,35,36,37]. We have also discussed current issues that have arisen because of the implementation of asynchronous duty cycles in opportunistic routing. The main contributions of this paper are summarized as follows:

- We highlight the main reasons behind energy drainage while applying asynchronous duty-cycled MAC functionalities to opportunistic routing protocols in WSNs;
- We have provided a review of the asynchronous duty-cycled MAC protocols that have been proposed up until the present time, and explain their data transmission procedure with an illustrative diagram. We have also discussed their main contributions and limitations regarding energy consumption;
- We have presented a literature review of opportunistic routing protocols and their evaluation, while explaining their base MAC mechanisms;
- We have focused on energy consumption, and have analyzed and compared the OR protocols based on their addressed challenges, deployed networks, base MAC functionalities, decision metrics, advantages and disadvantages.

## 3. Opportunistic Routing Protocol in WSNs

### 3.1. Concept of Opportunistic Routing in WSNs

The main structure of the OR protocol is completely different from conventional routing protocols. In traditional routing, the transmission path is static and fixed before the node starts transmission. On the other hand, in the opportunistic routing protocol, the next hop is decided dynamically after the packet is received by a neighbor node. In OR, the sender broadcasts a packet to a group of forwarders rather than to a single pre-selected node. Usually, these forwarder nodes are ranked on the basis of the routing metric. After the broadcasting of the data packet and its successful reception by the group of forwarders, a coordination protocol is run. This coordination is performed among the successfully received prioritized forwarders in order to select a unique forwarder node to continue the transmission. This process is repeated until the packet reaches its final destination sink. Hence, the basic operation of an opportunistic routing protocol can be divided into four parts:

- Selecting a prospective group of forwarders;
- Broadcasting data packets to the selected forwarders;
- Finalizing a unique forwarder using a coordination system;
- Forwarding the packet.

In 2005, Biswas and Morris described ExOR [4], wherein they proposed opportunistic routing in WSNs for the first time. Here, they fragmented data packets into small batches during transmission. Each batch had an identification number. The source node set the forwarder among its neighbor nodes and ranked them according to ETX (Expected Number of Transmissions) metrics. The closer the forwarder node was to its destination, the higher was the rank. Each node in the forwarder list managed a local batchmap. A batchmap is the list used for tracking the batch packet that has been forwarded by the node. Every time a node overhears a transmission, it compares its local batchmap with the packet’s batchmap, and if the higher ranked entry is found, it updates its local batchmap with that entry. ExOR implements the scheduled transmission of packets to ensure that only one node sends the packet at one time.

**Figure 3:**
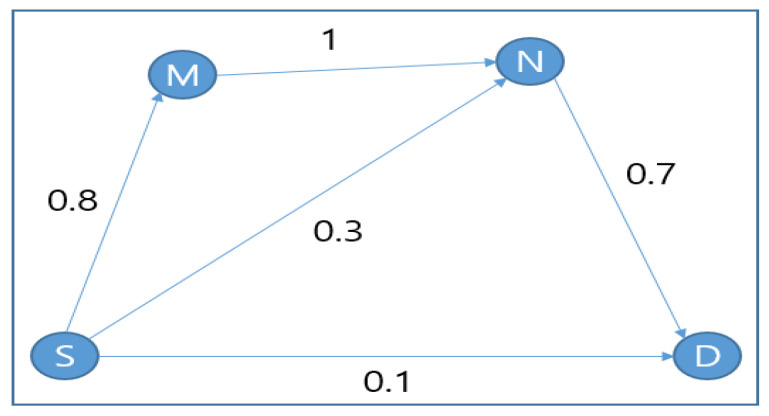
A network with the link delivery probability of each node pair [4].

In the network shown in Figure 3, there are four nodes connected to each other, with each given a link delivery probability. Consider a scenario where node *S* wants to send a packet to node *D*; node *S* therefore makes a forwarder list with *M*, *N* and *D*, where *D* is the highest priority. After receiving the packet, each node attempts to send an *ACK* (Acknowledgement). If node *N* does not hear the *ACK* from *D*, it will send its *ACK* embedded with its own sender *ID* as the highest forwarder. Then, node *M*, which would hear the *ACK* from *D*, would send its *ACK* embedded with *D*’s *ID* as the highest ranked node. Hence, node *N* will not forward the packet, as it knows through *M*’s *ACK* that the highest ranked node already obtained the data. ExOR showed improved throughput compared with conventional routing. However, it only works with the information available at the time of transmission, without considering update measurements. Hence, incorrect measurements can cause packet duplication. Even after the transmission, the nodes are concerned about the coordination by *ACK* among nodes, which causes overhead in the case of a dense network. Moreover, ExOR assumes that all nodes are active at all times. However, in a WSN where nodes are energy constraints, there is a critical bottleneck for load-balancing.

### 3.2. Concept of MAC Protocol for Maintaining Duty Cycle

**Figure 4:**
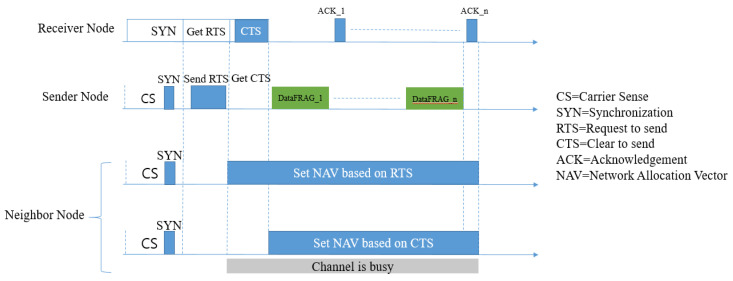
Packet transmission using S-MAC [1].

S-MAC [1] was the first MAC protocol that introduced a duty cycle mechanism into energy-constrained sensor nodes. Figure 4 shows the packet transmission procedure of S-MAC. This protocol was proposed by Ye et al. in 2002. The primary goal of S-MAC was energy conservation and self-configuration. In order to achieve these goals, the authors proposed three solutions:

- Nodes periodically enter into a sleep mode (periodic listening and sleeping);
- Neighbor nodes compose a virtual cluster in order to auto-synchronize in the sleep schedule (collision and overhearing avoidance);
- Apply ‘message passing’ to reduce contention latency for sensor network applications that require store-and-forward processing as data move through the network.

However, the main drawbacks of S-MAC are the fixed duty cycle (not tunable), and that it is energy inefficient. Moreover, the major problem was that in order to wake up at the same time as the neighbor nodes, this scheme used a synchronization process whereby several control packets would need to be broadcasted, which is a huge source of energy wastage. These were the reasons that motivated researchers to create protocols compatible with asynchronous duty-cycled WSNs.

## 4. Issues of Opportunistic Routing in Asynchronous WSN

While running the main operations of the opportunistic routing protocol, there are other related issues [3] concerning energy wastage that need to be taken into account, such as choosing efficient routing metrics, using an efficient scheme for prioritizing forwarder nodes, and a good coordination system for preventing duplicate transmissions and collisions. To handle these issues, some important properties need to be considered, such as the connectivity of the network, link status and communication overhead. In this section of our study, we address some challenging issues regarding the design and implementation of the opportunistic routing protocol, which are all related to energy consumption.

### 4.1. Multiple Sender

Data transmission can be divided into two categories based on initiation. It can be initiated by a sender or receiver. If the underlying MAC protocol is a sender-initiated protocol, then as soon as a sender wakes up, it will at first perform carrier sensing and start transmitting data packets or preambles if the channel is not busy. This leads to a critical issue. In an asynchronous duty-cycled wireless sensor network, one node does not know its neighbor node’s wakeup schedule, and if more than one node wakes up at the same time and starts initiating transmission, collision will occur at the receiver’s end. In the case of such a collision, the receiver will be unable to receive the packet and will cause senders to unnecessarily waste energy. On the other hand, the receiver also needs to keep its radio awake despite the occurrence of collision. Hence, in such a situation, both senders and receivers are bound to waste energy.

### 4.2. Multiple Receiver

In an opportunistic routing protocol, the sender broadcasts the packet opportunistically, so that if a potential forwarder wakes up earlier than other nodes and receives the packet, it can forward the packet successfully. However, because nodes wake up asynchronously and within a short time window, with a large number of nodes, there is a high probability that more than one node can wake up at the same time. As a result, when a sender broadcasts a packet, it is possible that more than one potential receiver can receive the packet and attempt to forward it upon successful reception. This problem is called a ‘multiple receiver’ problem where more than one forwarder exists for forwarding one data packet at a time. This will cause collision and unnecessary data retransmission, which will lead to more energy consumption.

### 4.3. Dynamic Route Selection

According to the opportunistic routing characteristics, packets will be transmitted opportunistically, and routes will not be pre-selected but will be chosen dynamically based on routing matrices. Because the next hop will be chosen dynamically, the sender nodes need to recalculate these routing matrices and choose potential forwarders in every hop repeatedly. However, this calculation is very costly and hence requires more time and resources. In addition, most of these calculations are performed based on assumptions and previously saved information, which may vary with topology changes and be more vulnerable given the asynchronous duty cycle behavior of sensor nodes. In the process of choosing a suitable routing path, all relative procedures lead to additional energy consumption.

### 4.4. Data Redundancy

One of the main reasons for unnecessary energy consumption is redundant data transmission or duplicate packet transmission. Because of the multiple receiver problem, one packet may be forwarded by multiple forwarders. As a result, there will be high levels of congestion in the dense network because of the same packet, which may run back and forth on the network and cause high energy wastage. Moreover, it will keep the channel busy and keep other potential forwarders waiting for the transmission of their new data packets. As a result, the length of the duty-cycle increases, which causes unnecessary energy consumption.

### 4.5. Control Packet Overhead

Since the next hop will be selected dynamically in opportunistic routing, it is necessary to collect link quality information and other useful information for the calculation of routing matrices in order to prioritize the forwarder list and select the highest potential forwarder. However, in order to collect such information for calculations, the sender needs to use a large number of control packets. These extra control packet overheads are a large source of energy consumption. Moreover, after ranking, the forwarder list sender also needs to let the forwarders know about their rank and act accordingly. As such, there is additional overhead for these control packets. For some protocols, this calculation is performed by the sender, whereas for other protocols, this is performed by the sink. Nevertheless, in both cases, the protocols need to send control packets in order to let the neighbor nodes know the information, which affects the network and increases energy usage.

### 4.6. Sender Waiting Time

Increasing the sender waiting time is also a large source of energy consumption in data transmission. Generally, in an asynchronous duty-cycled WSN, a sender node starts transmission without knowing the wakeup time of the receiver node. Hence, it keeps sending preambles until the receiver wakes up and receives the packet, and therefore this duration must be greater than the sleep interval. This will increase the energy consumption of the sender node, as well as keeping the channel busy so that, in the meantime, a potential transmission can take place between other node pairs. Moreover, in such situations, if a collision occurs and packets fail to reach their destination, all of these efforts and energy will have been spent in vain.

### 4.7. Receiver Waiting Time

As discussed in the previous sender waiting time issue, there is also an issue on the receiver side. On the receiver side, after a receiver wakes up and receives a preamble, it will wait for the actual data packet to arrive. However, even though it is already awake and ready to receive the packet, it has to wait longer for the actual data packet, which leads to additional energy consumption. Moreover, if a collision occurs and the receiver cannot receive the packet, then it will be a significant waste of energy.

### 4.8. Hidden Terminal Problem

In the hidden terminal problem, when two senders are unknown to each other because they are out of each other’s transmission range, while the receiver is in the range of both senders, there is a high probability that their packet transmission will collide with each other on the receiver end. In asynchronous duty-cycled WSNs, where nodes perform transmissions without prior knowledge, they are prone to collide with each other, whether it is a data packet or an acknowledgment packet. Hence, it is important to consider this issue in opportunistic routing with asynchronous WSNs, as this is a major issue that leads to unnecessary energy consumption.

### 4.9. Forwarder Cooperation

After a sender broadcasts a packet to its potential forwarder nodes, how the forwarder candidates collaborate with each other in order to perform the forwarding procedure smoothly must be evaluated. If they do not cooperate effectively, maintaining the traffic will be a hazard. If the forwarders who receive the packet successfully start forwarding after cooperating with each other, then the multiple forwarder problem, as well as the redundant packet transmission issue, can be resolved. As a result, the unnecessary energy consumption problem can be solved.

## 5. Evolution of Opportunistic Routing in Asynchronous WSN

Before we discuss the development of opportunistic routing protocols, it is essential to address the progress made by MAC protocols with asynchronous duty cycles in WSNs. Since the routing decisions of the opportunistic routing methodology are taken by the information that is collected by the underlying MAC mechanisms, it is important to perform a thorough review of the MAC protocols that can provide facilities to handle the problems faced in a MAC layer by an opportunistic routing protocol while working with asynchronous duty-cycled sensor nodes in a WSN.

### 5.1. Evolution of Asynchronous Duty-Cycled MAC Protocols

#### 5.1.1. B-MAC

In order to adapt asynchronous duty-cycling with WSNs, in 2004, Joseph et al. proposed a MAC protocol named B-MAC [28]. This protocol was specially designed for low-power sensor nodes with asynchronous wakeup–sleep behavior. This MAC protocol was built using a broadcasting mechanism; hence RTS (Request To Send)/CTS (Clear To Send)/ACK was absent here. This is why it is best suited for CSMA (Carrier Sense Multiple Access) behavior. The sensors using the B-MAC mechanism continuously send preambles until the receiver wakes up and starts receiving data, as the sender does not know about the receiver wakeup time.

**Figure 5:**
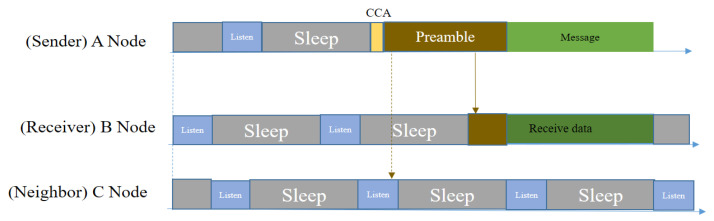
Packet transmission in B-MAC [28].

In Figure 5, node A sends a data packet to node B by first performing a CCA (Clear Channel Assessment), then sending preamble messages, and finally sending the data packet. Node B stays awake when it receives one or more beacon messages until the data transfer is completed. Node C will ignore the preamble and follow its sleep schedule even though it overhears the preamble, since that message is not destined for it.

However, this protocol has several drawbacks, even though it can reduce synchronization overhead. The sender needs to send a longer preamble, that is longer than the sleep interval of the node. By sending a longer preamble, it can ensure the successful transmission of data. However, for a small amount of data, this mechanism is costly and energy consuming. On the other hand, because it uses a broadcasting mechanism, there is a high probability of collision given the multiple packet problem. Because of the collision and absence of ACK, the sender will not be aware of transmission failure; hence the packet loss rate will be increased and, in the meantime, more energy will be drained. Moreover, the hidden node problem is not handled in this MAC protocol.

#### 5.1.2. Wise-MAC

In order to reduce the length of the wakeup preamble, El-Hoiydi et al. proposed the Wise-MAC [29] protocol in 2004. This protocol is also specifically designed for asynchronous duty-cycled sensor nodes. Wise-MAC is based on the preamble sampling technique (listening to the channel for carrier sensing). Even though it is made for asynchronous duty-cycled sensors (no synchronization scheme for the sleep–awake schedule), the sleep interval of all the nodes in the network is the same. It does not use RTS/CTS but uses ACK, which also carries information about the next wakeup time of the receiver. Only neighbors within a one-hop distance can know the wakeup schedule of the receiver node. Using the next wakeup time of the nearest receiver node, the sender node can send data at an exact time, with a short preamble added before the data.

**Figure 6:**
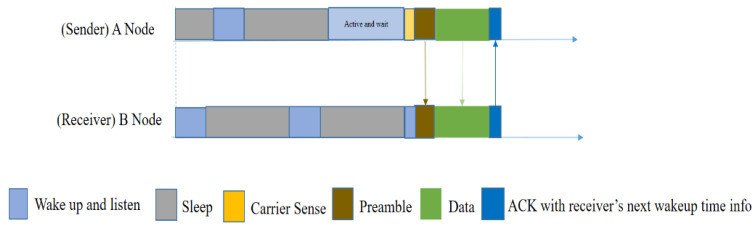
Packet transmission in Wise-MAC [29].

In Figure 6, node A has a packet to send, so it wakes up and waits until the receiver node wakes up. Node A starts sensing the carrier just before the receiver wakes up, and if the medium is idle, it then sends a small-length preamble. After obtaining a short preamble from A, node B will respond by an ACK that also contains the next wakeup time of node B. This information will be saved by the neighbor nodes that are in a one-hop distance from the B node, and can later be used if they want to send data to this specific receiver node B.

However, it does not provide a mechanism by which nodes can adapt to changing traffic patterns. Moreover, the authors did not address the hidden node problem because the RTS/CTS is absent. On the other hand, the first communication between two nodes will always be performed using a long wakeup preamble. Hence, the per-packet overhead decreases with increasing traffic, whereas in low traffic conditions, the per-packet overhead is high, which is again energy-consuming because most of the major applications of WSNs are related to environment monitoring, wherein nodes are idle most of the time and traffic is relatively low.

#### 5.1.3. X-MAC

X-MAC [30] is a design for asynchronous duty-cycled WSNs. The main feature of this protocol is that its preambles are shortened in order to reduce both the sender’s and the receiver’s waiting time. This mechanism reduces energy consumption for both the transmitter and the receiver. Small preambles are sent with a short pause so that the intended receiver can send an ACK reply in between, which can notify the sender to send data packets instead of more preambles. Because of preamble-ACK, the per-hop latency is reduced. X-MAC offers additional advantages such as flexible adaptation to both burst and periodic sensor data sources.

**Figure 7:**
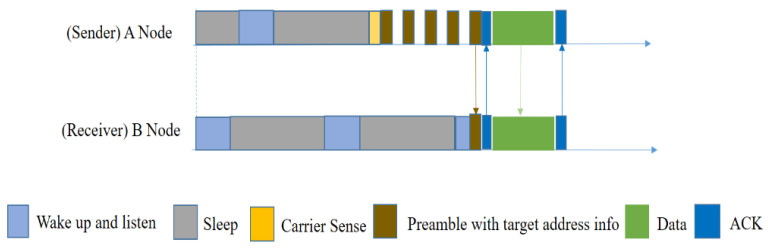
Packet transmission in X-MAC [30].

The packet transmission procedure in X-MAC is shown in Figure 7. Node A has a packet to send, and so it wakes up and sends short preambles with a pause between them. In each pause, node A waits for an ACK from receiver B. After preamble data is received by B, B will send a preamble-ACK. After receiving the preamble-ACK, A will stop sending the preamble and send the data packet instantly.

However, in a multi-hop network, if multiple senders send preambles at the same time, and all of them are out of each other’s transmission range, only having the same receiver will cause a hidden node problem. Then collision will occur at the receiver’s end, and it will be unable to send preamble-ACK, which will increase the sender’s waiting time and energy consumption as well.

#### 5.1.4. BoX-MAC

Two types of protocols were proposed in BoX-MAC: Box-MAC-1 and BoX-MAC-2 [31]. Rather than transmitting a wakeup preamble, as B-MAC does, BoX-MAC-1 continuously transmits the data packet directly. This continuously packetized wakeup transmission allows the BoX-MAC-1 receiver nodes to save energy by only staying awake for packets destined for them. This is a solution to the BMAC overhearing problem. BoX-MAC-1 is more efficient for use in high-volatility networks with little traffic.

On the other hand, the decoupling between packet size and receipt check duration allows BoX-MAC-2 to replace X-MAC’s dummy wakeup packets with the final deliverable packet. As a result, no handshaking is required in BoXMAC-2 to obtain the intended payload, and throughput can be nearly doubled compared to that of X-MAC. While both radios are awake, subsequent packets are delivered to the recipient at maximum throughput. In other words, multiple packets can be communicated bi-directionally after a single receive check detection. BoX-MAC-2 is more efficient in low-volatility networks with lots of traffic.

**Figure 8:**
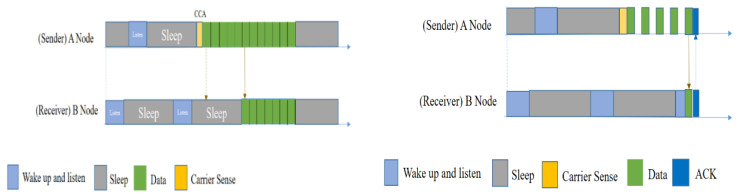
Packet transmission in BoX-MAC-1 (**left**) and BoX-MAC-2 (**right**) [31].

Using BoX-MAC-1 (Figure 8 (left)), node A continuously sends a data packet until a receiver wakes up and receives the data packet. Other nodes will avoid the packet if it is not meant to be transmitted to them, after the first packet they receive, and will go to sleep. On the other hand, in BoX-MAC-2 (Figure 8 (right)), node A has a packet to send. After waking up, it sends a data packet instead of a preamble with a pause within it. Each pause node waits for an ACK from the receiver. After data is received by receiver B, it will send an ACK.

However, two primary factors affect the performance of BoX-MAC protocols in a network: volatility and traffic. Sender-initiated low-power listening (LPL) protocols such as BoX-MAC are efficient in clean environments, but suffer from high power consumption due to false wakeups in noisy environments. In both of the Box-MAC protocols, the issue of the hidden node problem was not addressed.

#### 5.1.5. RI-MAC

The RI-MAC protocol [32] is a receiver-initiated MAC protocol, which is one of the most widely used protocols compatible with asynchronous duty-cycled WSNs. It has the potential to handle contending flows and thus burst traffic more efficiently and effectively. A sender using the RI-MAC protocol does not occupy the medium until the intended receiver is ready to receive the data. The receiver-initiated transmission makes RI-MAC more efficient in detecting collisions and recovering lost DATA frames. RI-MAC significantly reduces the amount of time a pair of nodes needs to occupy the medium for before they reach a rendezvous time for data exchange, compared to the preamble transmission in B-MAC and X-MAC. This short amount of medium-occupation time enables more contending nodes to exchange DATA frames with their intended receivers, which helps to increase the capacity of the network and thus the potential throughput. More importantly, this increase is adaptive; it lets a beacon serve as both an acknowledgment to previously received DATA, and as a request for the initiation of the next DATA transmission.

In RI-MAC, medium access control, among senders that want to transmit DATA frames to the same receiver, is mainly handled by the receiver. This design choice makes RI-MAC more efficient in detecting collisions and recovering lost DATA frames than B-MAC and X-MAC when the senders are hidden from each other, which is a common situation in multi-hop sensor networks. Together with the lower cost related to detecting collisions and recovering lost DATA frames, RI-MAC achieves higher power efficiency, especially when the network load increases. Following this concept, other recognized protocols have been proposed in the literature [38,39,40,41,42,43,44], which are all receiver-initiated MACs. An effective use of this protocol can be found in Zhang et al., 2019 [45]. Moreover, in order to evaluate the performance of this protocol, many survey studies [46,47,48,49,50] have been performed.

**Figure 9:**
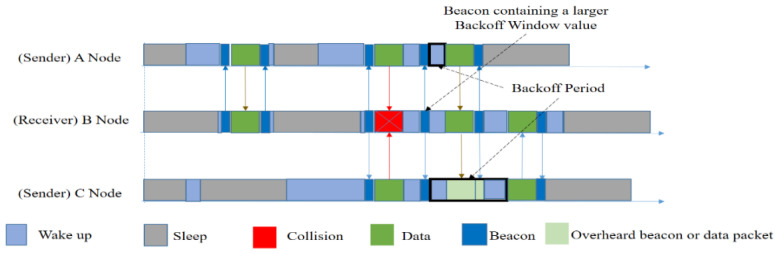
Packet transmission in RI-MAC [32].

In order to understand the actual data transmission procedure, we have provided a visual representation in Figure 9. Suppose that node A has a packet to send. It wakes up and silently waits for the receiver’s beacon. After obtaining the beacon, it will send data. Now, there are two senders, A and C, both of whom are waiting for B to wake up and are unaware of each other. Upon receiving B’s beacon, they both send data at the same time and collision occurs. Now B will send a beacon that will contain an extended back-off window size. Sender nodes will then choose a random back-off time to wait, and send the data again. In this way, RI-MAC handles its collisions.

However, when a sender has a frame to send, it immediately wakes up to wait for the receiver, leading to a longer sender duty cycle because of its idle listening for the receiver to wake up. The key idea of these receiver-initiated MAC protocols is very effective and powerful in terms of energy consumption. However, these protocols have a critical disadvantage when there are multiple senders and one receiver. In the case of multiple senders that are ready to send data frames, collisions may occur with very high probability because senders wake up at the same time as the receiver initiating. Moreover, only a single hop network was considered.

#### 5.1.6. DCM-MAC

DCM-MAC [33] was the first MAC protocol to introduce a multi-channel property to the asynchronous duty-cycling mechanism. DCM-MAC handles issues like decreasing synchronization overhead, allocating multi-channels dynamically, and managing multi-channel hidden node problems. It uses a receiver-initiated mechanism, and does cross channel handshaking to reserve a channel for transmitting data. This protocol provides three types of channels: (i) one control-channel (CC), (ii) multiple data-channels (DC) and (iii) one broadcast-channel (BC).

**Figure 10:**
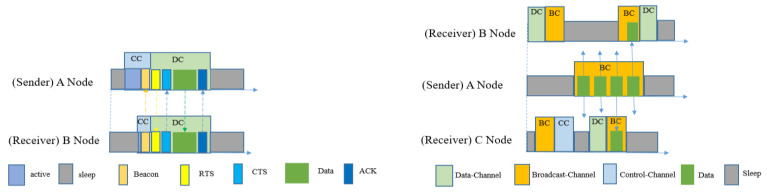
Packet transmission procedure of DCM-MAC in unicast (**left**) and broadcast (**right**) scenarios [33].

Figure 10 shows both the unicasting and broadcasting scheme used in DCM-MAC. In unicasting, if a sender node has any packet to send then it will wake up and silently wait for the receiver’s wakeup beacon. To the receiver’s beacon packet, the receiver will add the specific DC’s ID number where it intends to receive the data packet, and instantly switches to that channel. After receiving the beacon packet, the sender will switch to that specific DC and send RTS. After getting a CTS packet from the receiver, the sender will start sending the data packet and wait for the ACK. In order to avoid collision, it follows a random back-off procedure, the same as RI-MAC. DCM-MAC also provides a broadcasting mechanism whereby it assigns a BC for that procedure. If a sender has a broadcast-packet, then it will switch to BC and continuously send broadcast-packets N number of times. In order to receive the packet, the neighbor nodes will wake up once every N-1 time interval. The balance between energy consumption and latency is handled by the value of N. The simulations provided by [33] showed that this protocol improved energy consumption compared to RI-MAC in the case of unicast transmission. However, the energy efficiency of the broadcasting scheme using multi-channel with duty-cycling was not addressed.

#### 5.1.7. PW-MAC

PW-MAC [34] minimizes the energy consumption of sensor nodes by enabling the sender to predict the receiver’s wakeup time. It also introduces an efficient prediction-based retransmission mechanism to achieve high energy efficiency even when wireless collisions occur and packets must be retransmitted. It uses the basic concept of Wise-MAC, by getting the neighbor nodes’ wakeup–sleep timing from the ACK. Using a pseudo-random wakeup schedule rather than a fixed schedule, it avoids the possibility of neighboring nodes consistently waking up at the same time. This protocol also addressed the issues of single and multi-hop traffic flows, hidden node scenarios, and scenarios in which nodes have wakeup schedule conflicts, and the experiments showed that this protocol works better than previous protocols. PW-MAC improves protocols such as S-MAC and B-MAC because it uses pseudo-random schedules; thus, not all nodes will wake up and transmit at the same time, avoiding collisions. A node that has just woken up sends a short beacon so that other nodes know it is awake. A sender can then transmit a data packet and request more information from the receiver, such as current time and current seed, for the pseudo-random schedule used by the receiver. By using the seed in a linear congruential generator (LCG), the sender in PW-MAC can predict when a receiver wakes up; hence, the sender sleeps until a little bit before the receiver is awake, and it can effectively decrease the waiting time of the sender. PW-MAC reduces the energy consumption of sensor nodes, which is better than RI-MAC, by enabling sensors to predict the receiver’s wakeup time.

**Figure 11:**
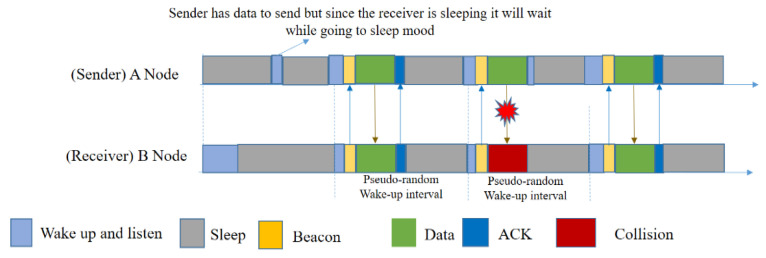
Packet transmission in PW-MAC [34].

Using PW-MAC (Figure 11), at first a sender, A, requests the prediction state of a receiver, B, and wakes up right before B does, after learning the prediction state of B. The prediction-based retransmission mechanism of PW-MAC enables A to detect the transmission failure and efficiently perform packet retransmissions.

However, there are hardware variations that generate errors in sender prediction. PW-MAC uses a “sender wakeup advance time”, a compensating value specific to every platform, including clock drift, operating system delay and hardware latency. The value helps to correct errors that each node can address when predicting a receiver wakeup time. The disadvantages of using PW-MAC include overhead created by beacons and idle listening, even if it is small compared to other protocols such as Wise-MAC, RI-MAC and X-MAC. This consumes a large portion of energy storage.

#### 5.1.8. MiC-MAC

MiC-MAC [35] is mainly a real-time, experimental-based, multi-channel and low-power listening MAC protocol. It uses the direct data frame as the wakeup preamble and a series of pseudo-random channel hopping. Overall, this protocol inherits ContikiMAC’s [51] design and adds the channel hopping functionality to it. It follows four step-by-step processes during data transmission: (i) accessing the medium; (ii) looking for the receiver’s wakeup time and available channel; (iii) transmission of data and acknowledgement; and (iv) handling collisions. In addition, senders keep track of communicating channels and wakeup times in order to use this information for later usage.

**Figure 12:**
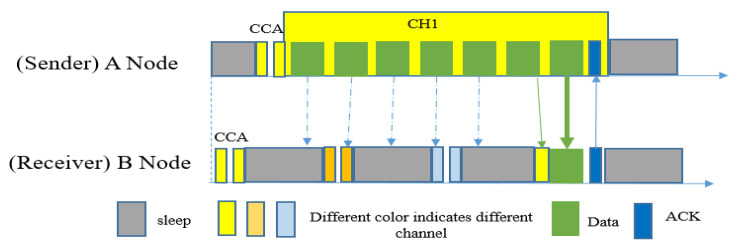
Packet transmission in MiC-MAC [35].

In this protocol [35], nodes wake up periodically and sense the channel with two short CCA signals. The nodes follow their own sequence to hop through all the available channels. If a sender has a packet to send (Figure 12), it will start sending direct data packets, instead of a preamble, through an available channel. It will continuously send data packets until it gets an ACK from the receiver side. It will send data packets for a maximum of three consecutive wakeup periods (because, in the example in Figure 11, there are three channels). In the case of a broadcast scenario, the sender nodes follow the same procedure, however they do not wait for ACK.

#### 5.1.9. RIX-MAC

The RIX-MAC [36] protocol combines the advantages of X-MAC [30] and PW-MAC [34]. X-MAC avoids collisions via other transmitter frames. In situations with multiple senders, TX-nodes (transmitting nodes) can prevent data collision by listening to the short preambles from other nodes and the early-ACK frame of a receiver (RX-node). PW-MAC reduces the TX-node’s power consumption by predicting the RX-node’s wakeup schedule. The wakeup state is divided into two periods: the Sched-wakeup (scheduled wakeup) and Synch-wakeup (synchronized wakeup) periods; the Synch-wakeup period is optional when a node wants to transmit data frames. RIX-MAC [36] transmits short preambles, as X-MAC does, and wakes up twice as much as PW-MAC. RIX-MAC handles the CA with a back-off time procedure. Even though senders cannot recognize each other, which is the case in the hidden node problem, involved neighbors can perform the same operation with a value in the duration field of the early-ACK frame. Although a random back-off decreases the probability of a collision, in the case of multiple senders, the RIX-MAC protocol uses a time-out approach when collision occurs. In the case of two or more TX-nodes that want to transmit data to an RX-node, the nodes wake up at the same time owing to the same information about the destination, try to transmit short preambles, and set the timer k just before transmission. If two or more of them choose the same random back-off, their short preambles collide. When short preambles collide, the RX-node is unable to decode and respond to them, and the TX-node continues to transmit short preambles until the RX-node’s early-ACK frame arrives, or the timer k expires. When the timer expires, the TX-nodes attempt retransmission with new random back-off values.

**Figure 13:**
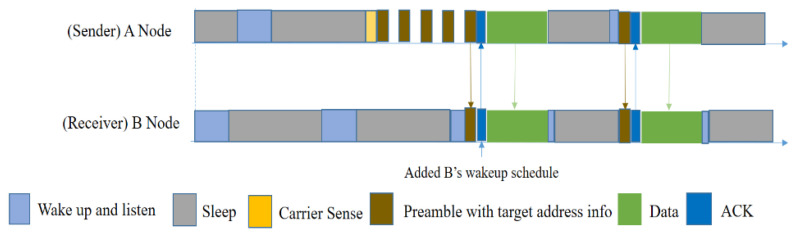
Packet transmission in RIX-MAC [36].

In RIX-MAC (Figure 13), node A has a packet to send; it therefore wakes up and sends short preambles with a pause after each preamble. During each pause, the node waits for a preamble-ACK from the receiver. After preamble data is received by receiver B, B will send a preamble-ACK with the information of its wakeup schedule. After receiving the preamble-ACK, the sender stops sending the preamble and sends the data packet instantly. Later, since A now predicts B’s next wakeup schedule, it will wake up before B and send a small preamble when B wakes up.

However, the TX-node not only has its own schedule, but also the temporary schedule of RX-node. The TX-node in RIX-MAC wakes up twice in one cycle. In the TX-node, the first wakeup occurs, according to its own schedule, to receive data frames (Sched-wakeup), and the second wakeup is used to transmit data to the RX-node (Synch-wakeup). This increases the active time and affects battery lifetime.

#### 5.1.10. TRIX-MAC

TRIX-MAC [37] is a novel MAC protocol for wireless sensor networks that uses a receiver initiation mechanism and tree topology structure for lower latency, lower energy consumption, and higher throughput. The operation of TRIX-MAC is substantially similar to that of RIX-MACs (Figure 13), which utilizes the receiver-initiated wakeup scheme as described in RI-MAC. TRIX-MAC reduces the number of overall control frames of the sender and receiver, and prevents the collision of frames in the situation of multiple children senders and a parent receiver. As a result, TRIX-MAC can limit energy consumption, unlike previous MAC protocols, such as X-MAC, PW-MAC and RIX-MAC. TRIX-MAC reduces the sender’s energy consumption with a smaller number of control frames by receiver-initiated wakeup scheduling, and removes the possibility of frame collisions by using the information of a tree structure.

However, this protocol only works on a tree structure. As such, the overhead of the control packet in the case of a topology setup, as well as at the time of topology change, is high, and will consume more energy, especially in a dense network. Moreover, the receiver node might wake up more frequently than the sender node, as it is difficult to predict the data transmission rates of individual sender nodes.

### 5.2. Evolution of Opportunistic Routing Protocols

#### 5.2.1. ORW

In 2012, Ghadimi et al. introduced ORW [11], which aims to provide a duty cycle-based WSN where, in order to reduce unnecessary energy consumption, nodes are continuously kept active and the sleep mode is asynchronous, whereas in ExOR [4], nodes are assumed to be active all the time. The concept of ORW is that the first node that wakes up will receive the packet and provide routing progress (ACK), and forward the packet on behalf of the source node. Like ExOR, ORW also uses ACK overhearing within the set of forwarders to avoid duplicate forwarding. Unlike ExOR protocols, where nodes worked with ETX [4,52,53], in ORW nodes use a routing metric called EDC (Expected Duty Cycle) [11,54]. First, the sender calculates its own EDC and probes its neighbors by adding its EDC value along with the packet header. Next, the neighbors accept the packet after waking up and updating the link quality estimation. Then, one unique neighbor forwards the packet while providing a routing progress. Finally, ORW handles multiple forwarding by observing some characteristics that rely on overhearing.

ORW does not depend on the tree structure, and the nodes are not synchronized either. The protocol assumes that the network uses asynchronous duty-cycling and very low power listening. Based on this model, the protocol tries to find a minimum-EDC forwarding node. As a result, in dense networks, this protocol shows a great improvement in energy efficiency and reduces transmission delay.

**Figure 14:**
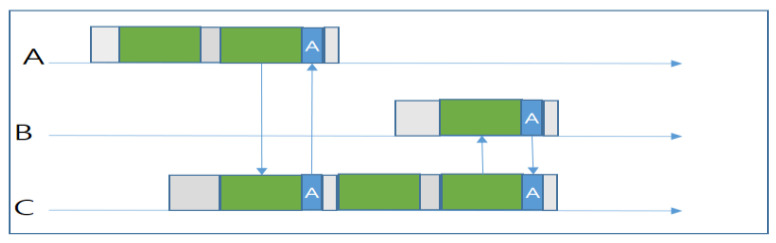
Packet transmission in ORW [11].

Suppose that node A has a weak link quality with node C but a strong link quality with B. According to traditional routing, node A would need to wait until node B wakes up and receives the packet. However, in the meantime, node C would wake up and hear the transmission of A. Hence, according to the ORW (Figure 14), since C has already overheard A’s transmission, it will receive the packet and provide an ACK as transmission progresses. Then, A will stop waiting for B and enter sleep mode. Now, C will keep probing for B to wake up, and transmit the packet successfully. In this way, ORW can reduce the sender’s waiting time as well as energy consumption.

However, the metric EDC used by ORW requires a recursive calculation, which takes a long time. Moreover, it does not consider the load-balancing problem. The node’s candidate set will change only when the network’s topology or link quality changes, which results in traffic load concentrating on nodes with a greater EDC. In addition, ORW does not limit the number of forwarders explicitly, so the multiple receiver problem is more likely to occur, which will cause packet duplication in the network. On the other hand, the overhead of link estimation is too high. ORW does not consider the impact of the independent duty-cycling of candidate nodes. In the estimation of the end-to-end (E2E) latency metric, only link quality is taken into account. ORW neglects the fact that a relay candidate node that wakes up more frequently should have a larger weight in determining the E2E latency.

#### 5.2.2. ORD

**Figure 15:**
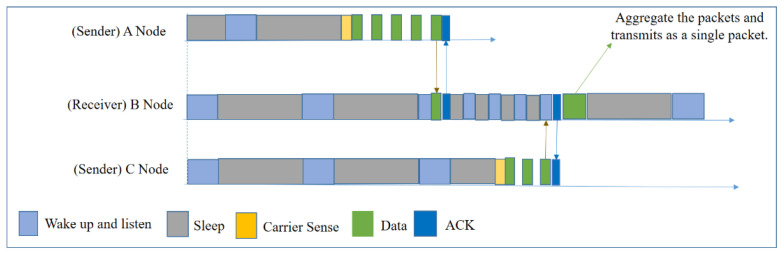
Packet transmission in ORD [12].

In 2014, Jungmin et al. proposed ORD [12], which is an improved version of ORW. The initial operation of ORD is similar to that of ORW. The core idea of this protocol is that if a forwarder node holds two data packets for the same destination, it will aggregate (Figure 15) the packets and transmit them as one packet in order to reduce the number of transmissions. To obtain more packets, the forwarder node waits for a specific amount of time. In this way, this protocol handles the tradeoff between energy consumption and latency. ORD can control the packet-holding time based on the application requirements. After choosing the forwarder set by EDC, the protocol decides the packet-holding time depending on the residual energy and hop count to the sink node.

In this protocol, the author used in-network aggregation to reduce the number of transmissions; however, in contrast, the forwarder node increases the frequency of the duty-cycle in order to obtain more packets, which will increase energy consumption considerably. Moreover, the packet holding time can increase the latency.

#### 5.2.3. ORR

In 2017, the same authors proposed another opportunistic routing protocol, named ORR [13]. It is also based on the ORW mechanism, but with a number of improvements. It uses a new metric called the ‘Forwarder Score’, which includes Residual Energy for calculating EDC. A node with a higher remaining energy is chosen as a forwarder node. ORR controls the number of forwarders based on the forwarding cost estimation. First, the ORR calculates the optimal number of forwarders based on the forwarding cost estimation. The optimal number of forwarders can differ according to the network type, so it needs to be calculated online during operation. Forwarding cost estimation considers the expected sender wait time and expected amount of redundant packets generated. Second, residual energy is considered when nodes select their forwarder sets. In ORW, forwarders are selected based on the expected wait time, so nodes with a large number of neighbors have a higher chance of becoming candidate forwarders. Thus, traffic load is often concentrated within a small number of nodes, draining their energy faster than other nodes. On the other hand, in ORR, nodes with a larger residual energy become selected forwarders more often.

However, the routing metric, ‘forwarder score’, is recursively computed at the sink. After computing, the sink broadcasts the forwarder score to all nodes, which may lead to congestion in a large network with a high density of nodes. In addition, the ORR updates the forwarder score periodically, and this updating’s overhead is expensive. On the other hand, to keep updating FS (Forwarder Score), residual energy is needed, and for this, each node broadcasts a control packet concerning their current energy status, which also adds to the load and contributes to extra overhead.

#### 5.2.4. MOR

**Figure 16:**
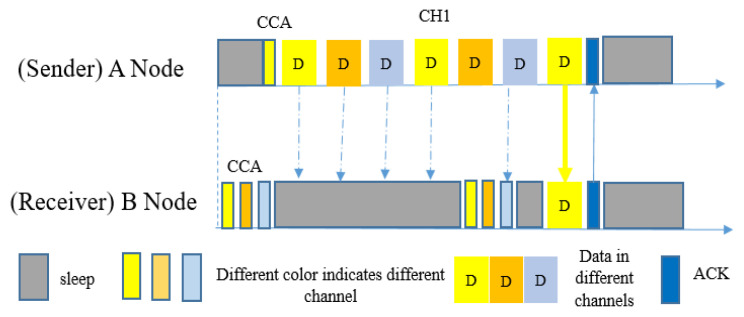
Sender- and receiver-initiated fast channel hopping scheme in MOR [14].

In 2017, Zhang et al. proposed Multichannel Opportunistic Routing (MOR) [14], a multi-channel opportunistic routing protocol for low-power asynchronous duty-cycled WSNs. In this protocol, the authors applied the concept of the opportunistic routing mechanism in the frequency domain, in order to improve the robustness of the network and the handling interference. The main concept of this protocol is as follows: The first node to wake up in the currently-transmitting channel will successfully receive the packet and give a routing progress update using the ACK packet. Later, this node will work as a potential forwarder for passing the packet to the next hop towards the sink. MOR has the advantages of using spatial and frequency diversities; if the suitable neighbor node for data forwarding is not available on a specific channel, then this protocol either uses a different channel or different available neighbor node to pass the packet, rather than wait longer. This protocol was practically applied on a real testbed to evaluate its performance. By increasing energy usage by only a small amount, MOR increased E2E reliability and decreased E2E latency, regardless of the channel interference. MOR uses two types of channel hopping: (i) sender-initiated slow hopping, and (ii) sender-initiated and receiver-initiated fast hopping. For the slow hopping scheme, MOR uses the MiC-MAC protocol, whereas in fast hopping both sender and receiver switch channels frequently within the same duty cycle, without waiting for the whole duty cycle (Figure 16). Usually, in other protocols, nodes re-transmit packets continuously on the same channel, but in MOR nodes re-transmit each time on different channels. However, in MOR, senders and receivers both use fast channel hopping, which is most of the time unnecessary and has a high energy cost.

#### 5.2.5. MORR

In 2018, Khan et al. improved the ORR [13] and proposed Modified Opportunistic Routing with Residual energy (MORR) [15]. They focused on the issues of network lifetime and load-balancing in WSNs. MORR makes use of the same methodology as ORR, but is more refined than ORR, in such a way that their proposed protocol considers channel interference and controls the number of forwarders by selecting nodes that have a minimum number of neighbors, while considering the maximum residual energy of forwarders, and controlling the number of transmitters as well. The contributions of this paper were the following. First, their focus was on channel interference for collision avoidance by selecting nodes with minimum neighbors and minimizing the duplicate packets. Second, they controlled the number of transmitters. Finally, they selected nodes with the maximum residual energy, in order to balance the consumption of energy and enhance the lifetime of the network. ORR and MORR have one major difference: MORR considers residual energy and minimizes the number of neighbors, while also considering the shortest distance to avoid collision and minimize duplicate packet transmissions. The forwarder set calculation is similar to the ORR, except that the forwarder with the minimum amount of neighbors is selected.

However, MORR does not consider duty cycle duration. To reduce the sender waiting time, the authors proposed a longer duty cycle, which is in opposition to reduced energy consumption. On the other hand, they select nodes with the minimum amount of neighbor nodes; in this case, there is a higher possibility that these nodes will drain energy earlier than the other nodes, because the same nodes will be selected multiple times.

#### 5.2.6. LORA

**Figure 17:**
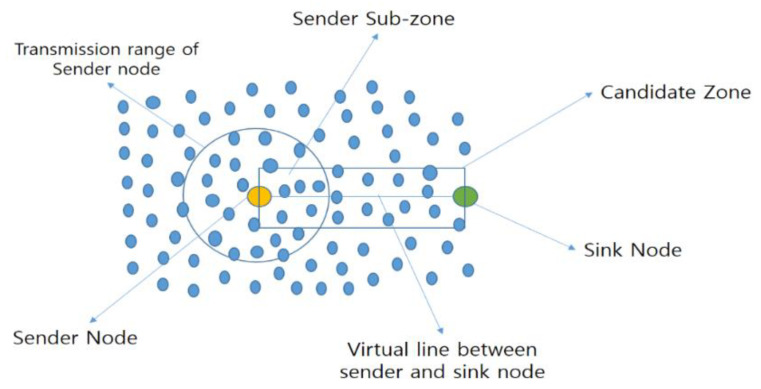
Packet transmission in LORA [16].

Recently, in 2019, Ammar et al. proposed Load-Balanced Opportunistic Routing for Asynchronous duty-cycled WSN (LORA) [16], an opportunistic routing protocol for balancing load issues in asynchronous duty-cycled WSNs. In this protocol, the authors applied significant changes and introduced new measurements for opportunistic routing. This protocol combines two main parts. First, each node defines a candidate zone (CZ) [16] using a regular geometric shape with four corners, as shown in Figure 17. Second, the candidates within the CZ are prioritized based on the OR metric. The OR metric is defined as the multiplication of four distributions: direction distribution, transmission distance distribution, perpendicular-distance distribution and residual energy distribution [16]. The authors focused on two major issues: the duplicate packet problem (on account of multiple forwarders) and sender waiting time (increased due to ACK collisions). The main objective of this protocol is to control the number of candidates in the network layer, and design an OR protocol that can counterbalance the sender waiting time with the multiple forwarder node problem. It mainly works with a duty cycle and a forwarder set. The size of the forwarder set is controlled by the network density.

In LORA, the authors identified that the energy required for routing a packet depends on three major components: (i) the transmission distance of each hop in the path; (ii) the length of the perpendicular distance from every selected forwarder node to the virtual line linking the source node and sink node; and (iii) the width of the direction angle between the sender node and the forwarder node, with respect to the location of the sink node. The nodes with smaller perpendicular distances are preferably selected as forwarders, because these nodes construct a straighter routing path. A smaller direction angle between the sender and forwarder means that the forwarder is closer to the sink, and potentially provides routing progress at a lower cost. A forwarder with a smaller transmission distance from the sender is preferable, as the energy consumption is related to transmission distance. Hence, the authors concluded that an energy-efficient path between the source and the sink nodes should select forwarders with smaller perpendicular distances, transmission distances and direction angles. They added the energy-balancing factor to avoid the energy depletion of some nodes earlier than others. Therefore, their routing metric (or link quality estimator) is defined as a multiplication of these four factors (four distributions).

However, in order to obtain this metric, the calculation cost is too high for every forwarder node because with an increase in network density, the number of multiple forwarders also increases. Moreover, they use a longer duty cycle in order to reduce the sender waiting time, which however increases the number of forwarders. For this reason, they focused on reducing the size of the forwarder set. On the other hand, the overhead of the calculations is higher and more time-consuming. In addition, this protocol did not consider the E2E latency. Compared with the previous OR protocols for asynchronous duty-cycled WSNs, LORA did not show any improvement in reducing sender waiting times.

#### 5.2.7. LEOR

In 2020, Omid et al. proposed Load-balanced and Energy-aware Opportunistic Routing (LEOR) [17], an opportunistic routing protocol for multi-channel asynchronous duty-cycled WSNs. The main purpose of this protocol is to increase network life-time by reducing energy consumption. The protocol works with scalability by considering network density, and remaining energy is used for the adaptive duty-cycling mechanism. LEOR handles data on two levels: the inter-nodes level and the inter-channels level. The adaptive asynchronous scheduling helps to balance energy load among different nodes. In addition, it uses multiple channels and ranks them based on channel utilization, which results in the avoidance of network interferences. Decreasing interferences, on the other hand, leads to a minimization of E2E delay and throughput. LEOR controls redundant data transmission by restricting forwarder selection-zone, which minimizes the number of forwarders.

It chooses a best forwarder node based on the distance from the receiver, the remaining energy of the forwarder and the number of its connections. LEOR works via two stages: the contention stage and the synchronization stage. When a sender has a packet to send, it will compete for access to the channel, and once it wins the channel it will synchronize with the selected channel and transmit the packet.

**Figure 18:**
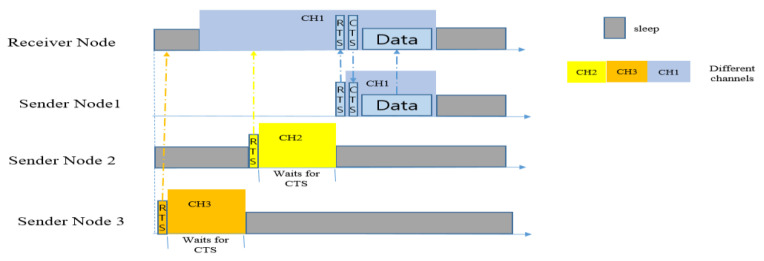
Contention and channel-hopping in LEOR [17].

Figure 18 shows the competition among three nodes on three different channels. Each node will wait for a specific amount of time to get a CTS reply from the receiver node. Once the winner node gets the CTS reply, it will instantly send the data packet and finish the transmission procedure. The simulation results provided in the paper in [17] were compared with MORR, and showed significant improvements.

### 5.3. Applications of Opportunistic Routing Protocols

#### 5.3.1. ODCR

In a recent publication, Al-kahtani et al. proposed Opportunistic Density Cluster-based Routing (ODCR) [55], an application of the opportunistic routing protocol for enhancing the energy efficiency and reliability of WSNs, using a density-based clustering mechanism which is specifically recommended for emergency and disastrous situations. Unlike the previously-mentioned protocols, in ODCR, the authors consider the nodes’ mobility features, and integrates edge computing with opportunistic routing. For data forwarding, ODCR uses a group of nodes (clusters) and also isolated nodes, where nodes in a cluster share the information among the cluster-members and an isolated node passes the data opportunistically to a group of potential neighbor nodes (potential nodes are selected based on their data delivery probability (DDP) value). Concerning the underlying MAC mechanism, this protocol used a self-assigned sender-initiated CSMA/CA mechanism. Like ORD, ODCR also aggregates data in order to reduce the number of transmissions. The experimental result provided in ODCR showed that this protocol outperforms LORA and the conventional WSN clustering protocols with regard to energy consumption, throughput and packet loss ratio.

However, in ODCR, since they have used DDP and MPC (Minimum Period Connectivity) as routing matrices and did not mention the duty-cycling of nodes, this indicates that the network nodes are always active and do not use the duty cycle mechanism. If duty-cycling is applied to ODCR, it is possible to extend the network lifetime remarkably. Moreover, it did not address the localization error calculation, which may occur because of node mobility. There is also the factor of data aggregation leading to increased latency, which can be a huge issue in emergency applications.

#### 5.3.2. AMOR

Lately, in 2020, Zhang et al. proposed Adaptive Motion-based Opportunistic Routing (AMOR) [56], an application of the opportunistic routing protocol that makes use of contact opportunities by carrying the data during the movement of the carrier. Hence, motivated by this mobility features of the carrier, this protocol offers an adaptive mobility-based opportunistic routing mechanism, which specifically works on opportunistic networks. Based on the data communication features, this protocol constructs a data forwarding priority model and defines the data transmission rules according to the nodes’ communication range. In addition, it proposed a tradeoff between the system overhead and transmission efficiency. In order to select a potential forwarder node, a free mobility level model is also proposed in this protocol. The simulation results provided with this protocol showed that this algorithm comparatively increases packet delivery ratio, and successfully decreases delivery latency. However, the authors did not discuss the energy consumption properties of this protocol.

## 6. Comparison Analysis and Research Challenges

### 6.1. Comparison Analysis

The using of multipath routing in distributing the energy load among available neighbor nodes is a very useful characteristic of opportunistic routing protocols. On the other hand, reducing the synchronization overhead in the MAC protocol is one of the most efficient ways to reduce energy consumption in duty-cycled sensor nodes. Hence, combining these two characteristics, the ORW was the first invention in this field.

However, even though this protocol was better than the conventional one, it has yielded issues related to energy consumption, discussed in Section 5, while working with the asynchronous duty-cycled WSN. Table 2 presents a short review of the milestone protocols proposed to date in the field of asynchronous WSNs. Table 2 illustrates which issues are addressed by these protocols and how they address the corresponding problems. The advantages and disadvantages of using these protocols are also presented in Table 2, so that, based on application requirements, they can be chosen whenever needed. However, from ORW to LORA, all of the opportunistic routing protocols described here are designed on top of a low-power listening Box-MAC protocol. This MAC protocol is an improved version of B-MAC and X-MAC, which increases the performance of the low-power listening asynchronous duty-cycled sensor nodes. Only LEOR has proposed its own MAC mechanism for communication. With its self-assigned MAC protocol, with multi-channel OR, it improves energy efficiency by 52% and 60% compared to MORR, in sparse and dense networks, respectively [17]. In Section 6, we have also provided a brief review of the development of asynchronous duty-cycle MAC protocols, where we have discussed the MAC protocols. ORW worked on a first-come-first-serve basis. The node that wakes up first can forward the packet, which will lead to multiple receiver and redundant packet transmission problems, because there is a high probability that more than one node will wake up at the same time and will listen to the transmission. ORD outperforms ORW because it performs additional calculations in order to shorten the competition of candidate nodes. In sparse networks, compared to ORW, the network lifetime is increased twofold, whereas in dense networks, it has been shown to increase by 76% [12]. However, ORD increases the frequency of the duty-cycle and holds packets for a long time, which affects both energy consumption and latency. ORR improved ORW and ORD by using a different routing metric called the Forwarder Score, which is evaluated based on the residual energy of neighbor nodes and the hop count to the sink node. ORR performs better in dense networks. In sparse and dense networks, the energy consumption in each node decreases by 5% and 72%, respectively [13]. As a result, it boosts the lifetime of the entire network tremendously. However, it has high calculation and overhead costs. In order to deal with these issues, MORR was later proposed. Compared with ORR, MORR decreases energy consumption by 85% and 83%, in sparse and dense networks, respectively [15]. Nevertheless, the authors did not consider the length of the duty-cycle, and instead used a longer active period, which is a major source of energy usage. In addition, choosing a small neighborhood with a minimum hop count will make the system choose the same nodes repeatedly, which will affect the load-balancing between nodes and create energy-holes in the network.

Recently, LORA [16] was introduced in the literature, where it presented a unique routing metric, which involves the multiplication of four distributions. Until recently, this protocol was the most efficient, and outperformed previously existing protocols for asynchronous WSNs. Simulations have so far been performed using a sparse network, the results of which have shown that LORA can reduce energy consumption by 30% and 35%, compared to ORW and ORR, respectively [16]. However, it also has significant issues that need to be addressed by future studies. All of the protocols described here have focused on load-balancing by controlling the size of the forwarder node group. Moreover, the simulations provided by most of the protocols are performed in both sparse and dense networks, except for LORA. LORA has been analyzed and simulated in a sparse network, but it is important to observe the behavior of the protocol in dense networks as well. Moreover, except for MOR, all the mentioned OR protocols are simulated in simulators, and the real-time experiments of MOR show that as the number of channels increases, the length of the duty cycle also increases, which leads to more energy consumption [14].

### 6.2. Research Challenges

#### 6.2.1. Multiple Sender

OR may frequently encounter a situation wherein a few senders try to transmit data at the same time, thus experiencing transmission collisions, which lead to energy waste. A conventional approach to deal with the situation is the receiver-initiated ORs [32,33]. This method is better for detecting collisions and recovering data packets, even though the latency is increased. In addition to the routing layer’s approach, we can consider a MAC layer’s approach to properly control the asynchronous duty-cycling (ADC) of the sender node. In other words, if the wakeup times of each sender node are not overlapped, the number of simultaneous senders is going to be minimized. PW-MAC may be an example, in that it used the information from the previous data transmissions in order to control its duty-cycle, which can avoid collisions.

#### 6.2.2. Multiple Receiver

Multiple Receiver, the so called forwarder set, is the most important issue in OR because the number of receivers directly corresponds to the number of senders in the next hop transmission of OR, which determines the degree of data redundancy. Several papers have been trying to optimally determine a forwarder set [11,12,13,14,15,16,17]. We can do research to make use of ADC in determining the forwarder set, because setting the nodes to different wakeup times may result in a different receiver set, or forwarder set. Investigating the relation between controlling the wakeup times of ADC and finding the optimal forwarder set is going to be one of the interesting research topics related to OR.

#### 6.2.3. Dynamic Route Selection

OR usually chooses its route dynamically among multiple potential routes to a sink. However, if the potential routes are too closely located, the benefits of dynamic routing are going to be reduced due to the transmission interference among routes. Thus, a good OR needs to form multiple potential routes not so close to each other. We can achieve this by properly selecting a good forwarder set. To this end, we can also consider to properly scheduled wakeup times of ADC in order to obtain a good forwarder set. It is going to be interesting to evaluate the effect of the ADC of nodes on the interference among the potential routes.

#### 6.2.4. Data Redundancy

OR intentionally permits data redundancy so as to make the data transmission reliable and robust. However, many uncontrolled redundant data packets, due to many uncontrolled forwarder nodes, causes a network to become congested, which leads to energy waste. In order to solve this issue, it is required to keep a good degree of data redundancy. Since the redundancy is affected by the forwarder set, which may be affected by the scheduling of the wakeup times of ADC, it is going to be interesting to investigate the effect of ADC on controlling the degree of data redundancy. For example, the references [11,12,13,14,15,16,17] have used sensor node ADCs in order to reduce the number of redundant packets.

#### 6.2.5. Control Packet Overhead

For the implicit sharing of routing information and estimating the link quality, sensor nodes must make the most use of overhearing the control packets broadcasted. However, the broadcasting of these control packets may be responsible for a large portion of energy consumption. Efficient overhearing of the control packets demands the timely wakeup of sensor nodes in ADC. However, none of the ORs have explicitly addressed the issues so far. We found that ORW [11] used continuous probing in order to estimate a link quality, which on the other hand consumes energy. In LORA [16], after consuming a quantity of energy, a node is supposed to broadcast a control packet so as to help other nodes calculate the routing metric again. It is going to be interesting to study the relation between minimizing the control packets and operating ADC.

#### 6.2.6. Sender Waiting Time

While the receiver-initiated MAC protocol [45] is proposed to mitigate the multiple receiver problem, it introduces another problem, that of sender waiting time. In the sender waiting time, a sender node should keep its radio active until a receiver’s beacon arrives. Thus, a longer sender waiting time may lead to energy waste. This issue may be tackled if the wakeup time of ADC is intelligently adjusted. PW-MAC is such an example wherein the receiver’s wakeup time is predicted in order to find a shorter waiting time, based on the previous wakeup times. It is going to be interesting to study how to determine a good sender waiting time based on the wakeup times of ADC.

#### 6.2.7. Receiver Waiting Time

Many OR protocols [11,12,13,14,15,16,17] in the asynchronous WSN belong to the sender-initiated MAC protocol. Because a sender in the MAC does not know the receiver’s wakeup schedules, the sender initiates a transmission by continuously sending a preamble or a data message, until the sender makes a rendezvous with at least a single receiver, repeating the wakeup and sleep cycle. However, if the sender’s data transmission is dynamic but rare, the receiver’s wakeup interval should be adapted according to the traffic pattern so as to save energy consumption. It is going to be interesting to study adapting the interval of ADC depending on the transmission pattern. Considering the use of multiple wireless channels with ADC may be another option [33].

#### 6.2.8. Hidden Terminal Problem

The hidden node problem [30] in multi-hop WSNs refers to a collision at a common receiver due to two different senders’ simultaneous transmissions located at the other side. These two senders cannot recognize each other’s transmission because they are out of one another’s transmission range. Applying good duty-cycling may be effective in mitigating this problem, because the duty-cycling may prevent the nodes from simultaneous transmission if the two nodes are set to not overlap their wakeup times. Thus, designing a duty-cycling for the hidden node problem seems to be interesting. On the other hand, a multi-channel procedure can also provide another good solution for the problem. By taking advantage of the multi-channels, DCM-MAC [33] could improve its performance in avoiding collisions.

#### 6.2.9. Forwarder Cooperation

An efficient cooperation among a group of forwarders can mitigate the aforementioned issues, such as multiple senders, multiple receivers and data redundancy. We can define efficient cooperation as a kind of transmission strategy negotiated by the forwarders; a node actually forwards a packet, while another node gives up the transmission, so as to achieve better energy consumption. However, there will be a tradeoff between the energy saving resulting from the cooperation, and the energy consumption due to the negotiation overhead. For example, ORW [11] used ACK packets for the cooperation between forwarders. Another interesting research topic will be the attempt to find a minimum negotiation overhead due to control packets, in order to have successful cooperation.

## 7. Conclusions

The scope of this study is to present a thorough review of the evolution of opportunistic routing protocols on top of ADCM in wireless sensor networks, and to outline their contributions to preserving the energy of battery-operated sensor nodes. We have presented a statistical graph in order to show the research trend for OR-ADCM, and provided an at-a-glance review table for highlighting our contribution compared to the previous related survey works. The concept of opportunistic routing and ADCM protocol have been explained here. We have pointed out the key issues for the energy consumption of OR in WSN. We have also provided a survey on the evolution of asynchronous duty-cycled MAC protocols in WSN, which will help the researchers to understand their main features and work-flow. In addition, a comparison analysis of the existing OR protocols with ADCM in WSN has been presented. Finally, the potential research challenges have been summarized.

## Figures and Tables

**Table 1:** Previous survey related works at a glance.

Papers	Survey Criteria	OR Protocols	ADCM Protocols	Partial Comparisons	Description
Ref. [18] 2015	Routing objectives, approaches, optimization tools.	√	X	√	Provided a comparative analysis that can give a clear view of the main features of the protocols and their contributions
Ref. [19] 2016	Performance of OR in different types of wireless networks.	√	X	√	Evaluate the performance of opportunistic routing protocols in other communication networks
Ref. [20] 2015	Various sleep–wake scheduling schemes with routing.	√	X	√	Describes various scheduling schemes with routing and their performance in a wireless sensor network
Ref. [21] 2015	Candidate selection processes and coordination schemes in OR.	√	X	√	Focused on the issue of reducing retransmission in order to prevent unnecessary energy consumption
Ref. [3] 2014	Overall routing issues of WSN.	√	X	√	Focused on all WSN issues and discussed the contribution of the opportunistic routing protocol to these issues
Ref. [22] 2009	Underlying access techniques of MAC protocols.	X	√	√	Focused on the energy efficiency, latency and throughput factors of WSNs and discussed the open research issues
Ref. [23] 2010	Issues of WSN related to MAC protocols.	X	√	√	Explained the MAC protocol functionalities in order to solve the WSN issues and pointed out hardware factors of the protocols
Ref. [24] 2013	Time management and the sender and receiver waiting time in MAC.	X	√	X	Classified the protocols and explained the course of motivation that drove the evaluation of the protocols
Ref. [25] 2014	Energy consumption factors of WSN MAC.	X	√	X	Explained how sensor nodes communicate with each other using their mismatched duty cycles in each MAC protocol
Ref. [26] 2015	Mode of operation in synchronous and asynchronous MAC.	X	√	√	Examined the protocols’ performances considering a linear/chain-type WSN
Ref. [27] 2017	Throughput, efficiency, stability, fairness, low access delay, low transmission delay and low overhead.	X	√	√	Presented the study of various WSN-specific MAC protocols based on various design factors
Our Survey	Unnecessary power consumption and load balancing factors of OR with ADCM in WSN	√	√	√	Based on load-balancing factors provided reviews on the evolution of ADCM and OR protocols and pointed out the issues while using both protocols together in WSN

**Table 2:** Short review on opportunistic routing protocols with asynchronous duty-cycled MAC in WSNs.

ORPA	Published Timeline	Addressed Issues	Routing Metric	Advantages	Drawbacks	Network	DC	Base MAC
ORW [11]	2012	Sender waiting time, multiple receivers, forwarder cooperation	EDC, PRR	Introduced new routing metric EDC; mechanism for choosing unique forwarders, increased throughput, decreased latency and energy consumption, flexible to link dynamics and changes.	Recursive calculation cost, ignores load-balancing issue, high control overhead, only topology change triggers recalculation of forwarder set.	Sparse to Dense (100~1000)	Asynchronous	BoX-MAC
ORD [12]	2014	Sender waiting time, energy consumption, number of transmissions, latency	EDC, Residual energy of each node, Hop count from the sink.	In-network aggregation, reduced number of transmissions, extended network lifetime, provides tradeoff between energy consumption and delay requirements.	Increased frequency of duty-cycle leads to more energy consumption, packet holding for aggregation causes increasing delay, high control packet overhead.	Sparse to Dense (100~700)	Asynchronous	BoX-MAC
ORR [13]	2017	Link quality estimation issues, multiple receiver problems, load-balancing issue, sender waiting time, average number of packet transmissions	Maximum residual energy, Forwarder Score	Control and optimization of forwarder nodes, balances energy consumption among forwarder nodes, increases network lifetime, improves the duplicate packet issue and sender waiting time effectively.	Sink periodically calculates FS and broadcast info, control packet overhead after each calculation, calculation costly, control overhead for residual energy information.	Sparse to Dense (100~700)	Asynchronous	BoX-MAC
MOR [14]	2017	Interference, multi-channel, channel hopping, sender waiting time, scalability	EDC, RPL integration	Robustness, decrease latency, usage of multiple channels, increased throughput, use frequency diversity, energy efficient	Channel occupation for a long time in slow-hopping and using all the channels at a time for a single packet transmission in fast-hopping.	Real-time experiment FlockLab testbed (30 TelosB nodes)	Asynchronous	MiC-MAC
MORR [15]	2018	Multiple forwarders, multiple senders, redundant packet transmission, link quality problem, load-balancing	FS, maximum residual energy, forwarders with minimum neighborhood, minimum hop count.	Uses mechanism to avoid collision and redundant packet transmission, number of forwarders is minimized effectively, minimizes latency and power consumption.	Considered a longer duty-cycle, repeatedly selecting same forwarders with small neighborhood decreases the scope of opportunistic routing.	Sparse to Dense (100~700)	Asynchronous	BoX-MAC
LORA [16]	2019	Sender waiting time, redundant packet transmission issue, load-balancing	Candidate Zone, Direction distribution, Transmission-distance distribution, Perpendicular-distance distribution, residual energy distribution	Local routing matrix calculation helps decreasing control overhead, no need for re-calculation unless topology change or energy degradation of nodes.	Probability of null subzone, inactive subzone and multiple receivers affect performance, high cost calculation and control overhead, E2E latency is ignored.	Sparse (100~200)	Asynchronous	BoX-MAC
LEOR [17].	2020	Sender-waiting time, duplicate packet transmission, multi-channel	distance from the receiver, remaining energy of the forwarder and the number of its communications	Uses multiple channel for minimizing interference, improved energy efficiency, throughput and delay	Receiver node’s duty cycle became longer because of adaptivity, only chooses the nodes far from sender and near to receiver, leads to high power transmission.	Sparse to Dense (20~500)	Asynchronous	Self-assigned MAC
